# Quality Comparison of 3 Tesla multiparametric MRI of the prostate using a flexible surface receiver coil versus conventional surface coil plus endorectal coil setup

**DOI:** 10.1007/s00261-020-02641-0

**Published:** 2020-07-21

**Authors:** T. Ullrich, M. D. Kohli, M. A. Ohliger, K. Magudia, S. S. Arora, T. Barrett, L. K. Bittencourt, D. J. Margolis, L. Schimmöller, B. Turkbey, A. C. Westphalen

**Affiliations:** 1 Department of Radiology and Biomedical Imaging, University of California, San Francisco, CA USA; 2 Department of Urology, University of California, San Francisco, CA USA; 3 Department of Diagnostic and Interventional Radiology, Medical Faculty, University Dusseldorf, 40225 Dusseldorf, Germany; 4 Department of Radiology and Radiological Sciences, Vanderbilt University Medical Center, Nashville, TN USA; 5 Department of Radiology, Addenbrooke’s Hospital, University of Cambridge, Cambridge, UK; 6 CamPARI Prostate Cancer Group, Addenbrooke’s Hospital, University of Cambridge, Cambridge, UK; 7 DASA Company, São Paulo, Brazil; 8 Department of Radiology, Fluminense Federal University (UFF), Niterói, Rio De Janeiro, Brazil; 9 Department of Radiology, Weill Cornell Medicine, New York, NY USA; 10 Molecular Imaging Program, National Cancer Institute, National Institutes of Health, Bethesda, MD USA

**Keywords:** MRI, Prostate cancer, Early diagnosis, Quality

## Abstract

**Purpose:**

To subjectively and quantitatively compare the quality of 3 Tesla magnetic resonance imaging of the prostate acquired with a novel flexible surface coil (FSC) and with a conventional endorectal coil (ERC).

**Methods:**

Six radiologists independently reviewed 200 pairs of axial, high-resolution T2-weighted and diffusion-weighted image data sets, each containing one examination acquired with the FSC and one with the ERC, respectively. Readers selected their preferred examination from each pair and assessed every single examination using six quality criteria on 4-point scales. Signal-to-noise ratios were measured and compared.

**Results:**

Two readers preferred FSC acquisition (36.5–45%) over ERC acquisition (13.5–15%) for both sequences combined, and four readers preferred ERC acquisition (41–46%). Analysis of pooled responses for both sequences from all readers shows no significant preference for FSC or ERC. Analysis of the individual sequences revealed a pooled preference for the FSC in T2WI (38.7% vs 17.8%) and for the ERC in DWI (50.9% vs 19.6%). Patients’ weight was the only weak predictor of a preference for the ERC acquisition (*p* = 0.04). SNR and CNR were significantly higher in the ERC acquisitions (*p*<0.001) except CNR differentiating tumor lesions from benign prostate (*p*=0.1).

**Conclusion:**

Although readers have strong individual preferences, comparable subjective image quality can be obtained for prostate MRI with an ERC and the novel FSC. ERC imaging might be particularly valuable for sequences with inherently lower SNR as DWI and larger patients whereas the FSC is generally preferred in T2WI. FSC imaging generates a lower SNR than with an ERC.

**Electronic supplementary material:**

The online version of this article (10.1007/s00261-020-02641-0) contains supplementary material, which is available to authorized users.

## Introduction

Multiparametric magnetic resonance (MR) imaging of the prostate has become integral to management of patients with suspected or known prostate cancer (PCA) [1, 2]. Prostate MRI was initially performed with an endorectal receiver coil (ERC) [3] because its proximity to the gland increased the signal-to-noise ratio (SNR) of the acquired images. The higher SNR could be exploited to increase spatial and/or temporal resolution [4, 5], possibly improving clinical performance [6–8].

However, ERCs entail several disadvantages such as increased costs, examination time, and discomfort for patients [9, 10], possibly compromising compliance. ERCs can also induce severe signal inhomogeneities [11] and artifacts because of their non-uniform reception profile and/or poor positioning. Finally, ERCs can cause anatomical distortion of the gland or even stimulate intestinal peristalsis and thus motion artifacts.

ERCs were previously considered indispensable when patients were scanned using 1.5 T MR scanners. However, several recent studies revealed comparable image quality and similar prostate cancer detection rates with or without ERCs using modern 3 T MR scanners and surface pelvic phased-array receiver coils [10, 12–17]. One potential limitation of prior studies is that all radiologists comparing the different coil setups came from the same or closely connected institutions, which may have influenced their quality assessment in favor of the routinely utilized technique in these institutions [9, 10, 13].

Recently, novel surface coil array designs have been developed that include flexible surface coils (FSC) that can conform to the perineum, thus minimizing the distance to the prostate.

In this multicenter study, we quantitatively and qualitatively compared the quality of 3 Tesla magnetic resonance imaging of the prostate acquired with the novel FSC and with a conventional ERC in T2-weighted images (T2WI) and diffusion-weighted images (DWI), since these are generally considered to be the most important sequences for prostate MR image interpretation.

## Materials and methods

### Study design and population

This retrospective, cross-sectional, HIPAA-compliant study was approved by the institutional review board with a waiver for signed consent.

Images from 150 men were selected from a pool of 1200 consecutive patients who underwent multiparametric MR imaging of the prostate between June 15, 2017 and April 14, 2019. The original pool of 1200 patients was divided into two groups. Group A (300 patients) consisted of patients who underwent MR imaging using the FSC. Group B (900 patients) received routine scans with an ERC.

A smaller, matched subset of these groups was chosen for study as follows: First, 50 men were randomly selected from group A. These patients were sequentially matched with 100 patients from group B at a 1:2 ratio by bodyweight (<70, 70–80, 81–90, 91–100, >100 kg), age (<55, 55–60, 61–65, 66–70, 71–75, >75 years), prostate volume (<30, 30–40, 41–50, 51–70, >70 ml), and prostate-specific antigen (PSA) range (<4.00, 4.00–10.00, >11.00 ng/ml) in that particular order, to ensure similar conditions. These matched parameters were chosen to minimize potential confounding factors, for example, distance between the surface coils and the prostate. Previous biopsy status, histopathological results, and treatment history were also retrieved from the electronic medical records. All data was collected by one of the authors (TU).

### Imaging protocol and coil

Imaging protocols were in accordance to the PI-RADS v2 guidelines.

#### Group A

Images were acquired on a 3T MR scanner (Premier Signa, GE Healthcare) using a body coil for excitation and a flexible surface phased-array coil (Air, GE Healthcare) for reception.

Thin-section high-spatial resolution axial T2-weighted 2D Fast Spin Echo (FSE) MR images of the prostate and seminal vesicles were obtained using the following parameters: Field-of-view (FOV), 18 × 10 cm; repetition time (TR)/effective echo time (TE), 9225/120; echo-train length, 30; section thickness, 3 mm; no intersection gap; acquisition matrix, 320 × 300 (512 × 512 interpolated reconstruction); frequency direction, anteroposterior, flip angle, 160°; and 1 signal excitation. The final voxel size was 0.56 mm × 0.6 mm × 3 mm (interpolated voxel size 0.35 mm × 0.35 mm × 3 mm).

Diffusion-weighted images (DWI) were obtained using a spin-echo echo planar imaging (SE-EPI) acquisition using b values: (0, 600) s/mm^2. Other acquisition parameters were as follows: FOV, 18 cm; TR/TE, 4500–4621 ms/minimum; thickness/gap, 3 mm/0 mm; acquisition matri× 100 × 64; acceleration factor, 2; number of excitations, 6.

#### Group B

Images were acquired on a 3 T MR scanner (Discovery MR750, GE Healthcare) using a body coil for excitation and a pelvic phased-array coil together with an inflatable endorectal coil (E-Coil, Medrad) filled with air for reception.

Thin-section high-spatial-resolution axial T2-weighted 2D FSE MR images of the prostate and seminal vesicles were obtained using the following parameters: FOV, 18 cm; TR/effective TE, 5600–7400/99–114; echo-train length, 16; section thickness, 3 mm; no intersection gap; acquisition matrix, 384 × 384 (512 × 512 interpolated reconstruction); frequency direction, anteroposterior, flip angle, 111; and 1 signal excitation. The final voxel size was 0. 47 mm × 0. 47 mm × 3 mm (interpolated voxel size 0.35 × 0.35 × 3 mm).

Diffusion-weighted images (DWI) were obtained using a spin-echo echo planar imaging (SE-EPI) acquisition using *b* values: (0, 600) s/mm^2^. Other acquisition parameters were as follows: FOV, 18 × 10 cm; TR/TE, 4725 ms/minimum; thickness/gap, 3 mm/0 mm; acquisition matrix 128 × 64; acceleration factor, 2; number of excitations, 6. Signal non-uniformity due to the presence of an ERC was corrected using the available coil-correction software [18].

Both protocols also included sagittal and coronal T2-weighted 2D FSE images, T1-weighted images, dynamic contrast-enhanced spoiled gradient echo images, and, in some cases, spectroscopic images, T2-weighted 2D PROPELLER images, and T2-weighted 3D FSE images, but these were not reviewed in this study. Both protocols also included higher *b* value DWI (group A: 0, 1000 s/mm^2^; group B: 0, 1350 s/mm^2^), extrapolated high *b* value images (1400 s/mm^2^ and 2000 s/mm^2^), and calculated apparent diffusion coefficient (ADC) maps, all of which were not reviewed in this study. All T2-weighted images were obtained prior to the injection of gadolinium.

### Quantitative image assessment

The SNR and contrast-to-noise ratios (CNR) were measured using our institutional Picture Archiving and Communication System (Impax 6, Agfa Healthcare).

Regions of interest (ROI) were systematically drawn on T2WI at approximately the same location in all patients. This was done to minimize the impact of the distance between the ERC and the ROI in group B. The signal intensities (SI) of the whole prostate, peripheral zone (PZ), and transition zone (TZ), as well as of urine in the bladder were measured in all patients. The standard deviation (SD) of the SI of the urine was defined as image noise, assuming a homogenous composition and, therefore, SI of the urine.

In patients with histologically proven prostate cancer, the signal intensities of the MRI lesions that were positive on MR-TRUS guided fusion biopsy were also measured.

Calculation of SNR and CNR was performed as follows [19, 20]:

$${\text{SNR}} = \frac{{{\text{tissue signal intensity}} }}{{{\text{image noise}} }} = \frac{{{\text{SI}}\left( {\text{tissue}} \right)}}{{{\text{SD }}\left( {\text{bladder}} \right)}}$$

$${\text{CNR}} \left( {\text{tumor}} \right) = \frac{{{\text{tumor signal}} - {\text{tissue signal}}}}{{ {\text{image noise}}}} = \frac{{{\text{SI }}\left( {\text{PCA}} \right) - {\text{SI }}\left( {\text{BT}} \right)}}{{ {\text{SD }}\left( {\text{bladder}} \right)}}$$

$${\text{CNR}} \left( {{\text{TZ}}/{\text{PZ}}} \right) = \frac{{{\text{PZ signal}} - {\text{TZ signal}}}}{\text{image noise}} = \frac{{{\text{SI }}\left( {\text{PZ}} \right) - {\text{SI }}\left( {\text{TZ}} \right)}}{{{\text{SD }}\left( {\text{bladder}} \right)}}$$

Note – SI = signal intensity, SD = standard deviation, BT = benign tissue in the respective intraprostatic zone, PCA = prostate cancer, PZ = peripheral zone, TZ = transitional zone.

### Qualitative image assessment

De-identified T2-weighted and diffusion-weighted MRI sequences were retrieved from PACS and transferred to a web-based annotation platform (MD.ai, New York, NY) that was utilized to show images to readers [21]. Aside from de-identification, no other modifications were made to the DICOM images.

Six radiologists from different imaging centers in four countries and at least 6 years of experience reading prostate MR images independently assessed multiple pairs of images. 3/6 readers evaluated all cases, whereas the other three readers assessed half of the cases due to time constraints. Each pair consisted of images of an examination from group A (FSC) and one of its matched cases from group B (ERC) combined into a single patient with two series so that they could be compared side-by-side in a single viewer. Further, paired images consisted of either T2WI or DWI. Thus, 200 pairs of sequences were available for comparison. Furthermore, each group A (FSC) examination was compared to two different group B (ERC) examination. As readers were not aware that each group A image was going to be shown twice, these were used as an internal control of consistency of assessment. The order in which pairs were presented was randomly determined. For each pair, readers chose which set of images (right or left) was generally preferred (better quality). Next, readers assessed each individual sequence’s general image quality, as well as its delineation of the prostate boundary and differentiation of the peripheral from the transition zone using a 4-point scale: 1=excellent, no need to rescan; 2=adequate, good to interpret but could rescan to improve quality if easy; 3=marginally acceptable, best to rescan but would interpret if rescanning is difficult; 4=not acceptable, must rescan.

Readers also evaluated image distortion, motion artifacts, and other artifacts on 4-point scales: 1=none or minimal, no impact on interpretation; 2=moderate, minimal impact on interpretation; 3=pronounced, limits interpretation; and 4=marked, precludes interpretation.

Readers did not have access to any medical history. All answers were collected using a web-based survey platform (REDCap, Vanderbilt University, Nashville, Tennessee).

### Statistical analyses

Descriptive statistics and the corresponding measures of dispersion were used to summarize the population characteristics. For the objective image assessment, the Mann–Whitney *U* test (MWU) was used to test non-parametric data. To clearly separate the two sources of variation in the subjective image analysis in this study, (a) the slides presented to the radiologists and (b) the individual radiologists who rated the slides, we used a two-level analysis. First the data for each radiologist were summarized, then we summarized the data across radiologists using a meta-analysis with a random-effects model. Cohens’ kappa coefficient was calculated as measure of intrareader agreement, i.e. consistency of the given single scores for duplicate MRIs. Agreement was defined as almost perfect (*k* > 0.81), substantial (*k* = 0.61–0.80), moderate (*k* = 0.41–0.60), fair (*k* = 0.21–0.40), and poor (*k* ≤ 0.20) [22]. To compare the mean scores assigned to the two different imaging techniques we used the paired Wilcoxon rank-sum test and the Mann–Whitney *U* test. We used multivariate logistic regression to determine if patients’ age (continuous variable), prostate volume (continuous variable), bodyweight (continuous variable), PSA value (continuous variable), prior treatment (categoric variable), or readers’ number of years of experience (ordinal variable), and experience with ERC (categoric variable) were predictors of the choice for FSC or ERC images. Statistical analysis was performed using IBM SPSS^®^ Statistics (Version 22, IBM, Germany). All tests were two tailed, and a 5% level of confidence was considered statistically significant.

## Results

### Patients

Approximately half of patients in group A (26/50 patients, 52%) and about two-thirds of patients in group B (69/100, 69%) had biopsy-proven PCA at the time of the scan, whereas PCA was suspected in the remaining cases due to elevated PSA. Gleason scores (GS) of men with known PCA in group A were 3+3 (14/50, 28%), 3+4 (8/50, 16%), 4+3 (2/50, 4%), 4+4 (1/50, 2%), and 4+5 (1/50, 2%). Gleason scores of men with known PCA in group B were 3+3 (47/100, 47%), 3+4 (16/100, 16%), 4+3 (5/100, 5%), and 4+4 (1/100, 1%). Follow-up biopsy revealed another 10 PCA cases in group A (GS 3+3, 3/50, 6%; GS 3+4, 3/50, 6%; GS 4+3, 3/50, 6%; GS 4+4, 1/50, 2%) and another 14 PCA cases in group B (GS 3+3, 4/100, 4%; GS 3+4, 6/100, 6%; GS 4+3, 1/100, 1%; GS 4+5, 3/100, 3%).

In group A, 20/26 (77%) men with known disease were on active surveillance, six had received prior treatment as follows: permanent prostatic implant brachytherapy (3/26, 11%), external beam radiation therapy (1/26, 4%), and focal cryoablation (2/26, 8%). In group B, 52/69 (75%) men with known disease were on active surveillance, 17 had received prior treatment as follows: permanent prostatic implant brachytherapy (8/69, 12%), external beam radiation therapy (3/69, 4%), focal cryoablation (4/69, 6%), and androgen deprivation therapy (2/69, 3%).

Patients in group A and group B did not differ significantly in bodyweight, age, prostate volume, and PSA range (Supplementary Table 1).

### Quantitative image assessment

Table 1 shows that the SNRs of the whole prostate, peripheral zone, transition zone, and PCA lesions were significantly higher when T2WI were acquired with the ERC (*p*<0.001). The CNR discerning PZ from TZ was also significantly higher when images were acquired with an ERC (*p*<0.001). The CNR discerning PCA lesions from benign prostatic tissue were also higher in T2WI acquired with an ERC, but the difference was not statistically significant (*p*=0.1).

**Table 1:** Signal-to-noise ratios and contrast-to-noise ratios using either the flexible surface coil or the endorectal coil in the whole prostate gland, the peripheral zone, the transition zone, and in biopsy-proven prostate cancer lesions

Signal-to-noise ratios (SD)	FSC	ERC	*p* value^a^
Whole prostate	33.99 (11.41)	59.30 (36.46)	<0.001
Peripheral zone	33.99 (10.84)	76.74 (51.15)	<0.001
Transition zone	34.94 (12.12)	52.49 (42.00)	<0.001
PCA lesions	44.80 (15.49)	83.11 (44.96)	<0.001
Contrast-to-noise ratios (SD)	FSC	ERC	*p* value^a^
PCA—Prostate	8.85 (6.41)	18.82 (28.18)	0.1
PZ—TZ	− 0.94 (7.43)	24.25 (17.96)	<0.001

*SD* standard deviation; *FSC* flexible surface coil; *ERC* endorectal coil; *a* Mann–Whitney *U* test; *PCA* biopsy-proven prostate cancer lesions; *PZ* peripheral zone; *TZ* transition zone

### Qualitative image assessment

#### General preference

**Fig. 1:**
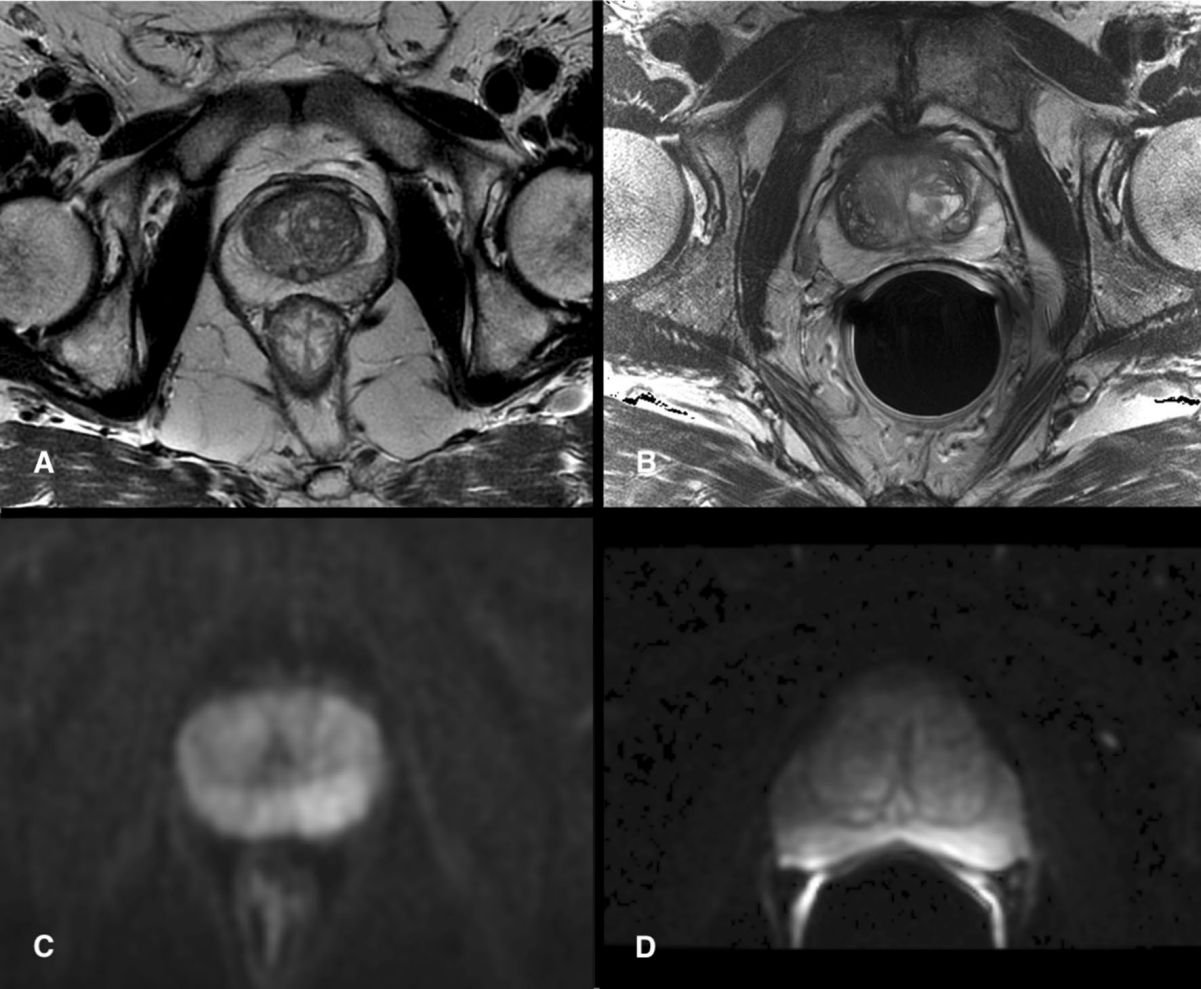
Four random images of MRI data sets that were presented to the readers on a web-based annotating platform. **a** A T2-weighted image acquired with the flexible surface coil in comparison to a T2-weighted image acquired with an endorectal coil (**b**). **c** Illustrates a trace diffusion-weighted image (tDWI) acquired with the flexible surface coil in comparison to tDWI acquired with an endorectal (**d**)

Figure 1 illustrates exemplary paired T2WI and DWI data sets as presented to the readers.

Table 2 shows that the pooled choice for the better overall image quality was not significantly higher for one coil setup compared to the other when evaluating the entire set of images. Yet, when the sequences were evaluated individually, there was a slight pooled preference for the T2WI obtained using the FSC and for the DWI obtained using the ERC. Supplementary Table 2 details the data for all individual readers.

**Table 2:** Pooled overall preference for MRI examinations and sequences performed with the flexible surface coil or the endorectal coil

	FSC	Either	ERC
Entire study	262 (29.1)	329 (36.6)	309 (34.3)
T2WI	174 (38.7)	196 (43.6)	80 (17.8)
DWI	88 (19.6)	133 (29.6)	229 (50.9)

*FSC* flexible surface coil; *ERC* endorectal coil; *T2WI* T2-weighted image; *DWI* diffusion-weighted image; *either* no preference for one or another set of images. Numbers are counts with percentages in parenthesis

**Fig. 2:**
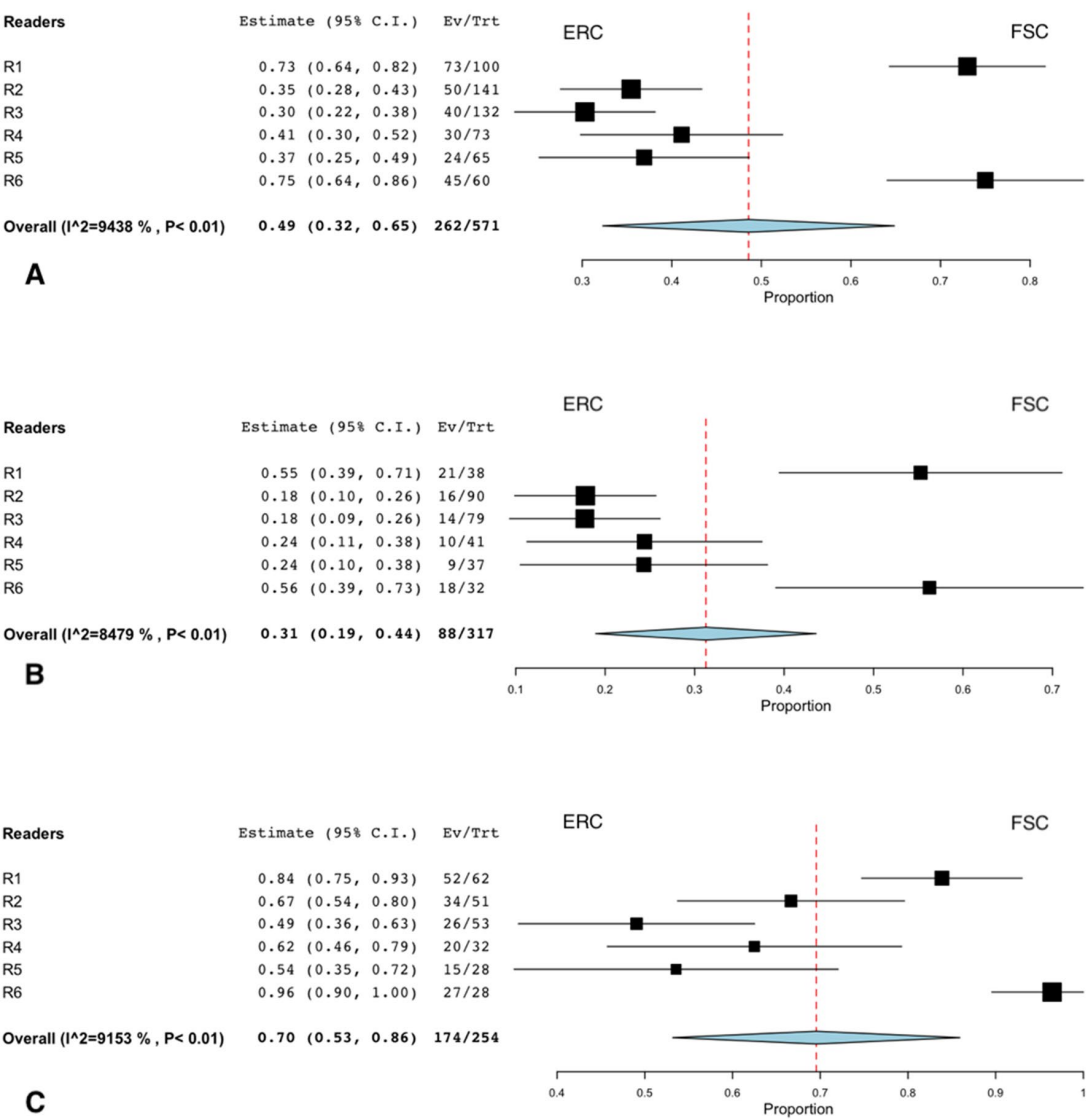
Forest plots show individual and pooled preferences for examinations performed with the flexible surface coil (FSC) or the endorectal coil (ERC). The first plot (**a**) summarizes the preferences for both sequences combined. The other two figures summarize the data for DWI (**b**) and T2WI (**c**) alone. Each plot excluded cases in which readers had no particular coil preference (“Either”). The summary estimate (diamond) indicates that the pooled preference for both sequences combined was balanced although readers had strong individual preferences for images obtained with one or other coil setup (**a**). Readers generally preferred the ERC for DWI (**b**) and FSC for T2WI (**c**). Dashed line denotes summary measure. *CI* confidence interval, *Ev/Trt* number of preferences for FSC if there was a preference, *R* reader, *p p* value

Figure 2 shows that each reader had strong individual preferences, favoring one of the two techniques in almost twice as many cases as the other. Two readers chose the FSC acquisition as the better overall image quality more often (36.5% and 45%) than the ERC acquisition (13.5% and 15%). Four readers preferred the ERC acquisition (range, 41–46%) over the FSC acquisition (range, 20–30%). The pooled summary estimate shows no significant difference in the overall preference for both sequences combined. If considering only T2WI, there was a pooled preference for the FSC acquisition (38.7%) over the ERC acquisition (17.8%). However, there was a pooled preference for the ERC acquisition (50.9%) over the FSC acquisition (19.6%) when DWI is evaluated alone. All readers tended to prefer the same technique, FSC or ERC, for T2WI and DWI if images were from the same patient. If the FSC acquisition was preferred on T2WI, the same coil or either coil was preferred in DWI in 63%. If the ERC acquisition was preferred on T2WI, the same coil or either coil was preferred in DWI in 80%.

#### Image quality scores

**Fig. 3:**
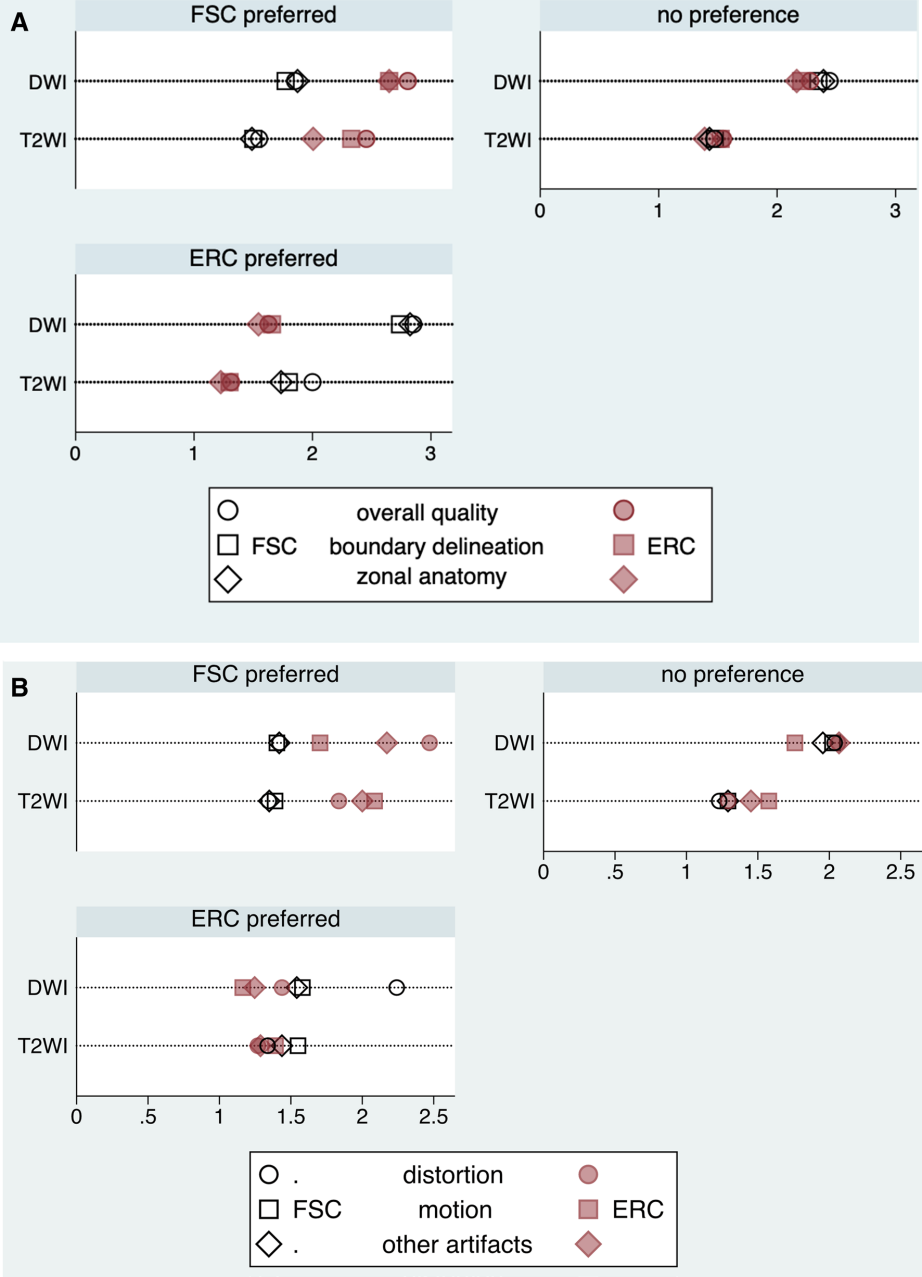
Dot plots depict flexible surface coil (FSC) and endorectal coil (ERC) T2WI and DWI subjective assessment mean scores stratified by readers’ preferences. **a** Summarizes general image quality, delineation of the prostate boundary and differentiation of the peripheral from the transition zone. A lower score indicates better perceived performance. **b** Depicts image distortion, motion artifacts, and other artifacts. A lower score indicates better perceived performance

Figure 3 and Table 3 show that when readers had a general preference for one of the two techniques, they assigned better mean scores for every item asked subsequently. Yet, the differences were not statistically significant for “motion artifacts” in DWI when FSC was initially preferred (*P*=0.11) and in T2WI when ERC was preferred (*P* = 0.47), and “distortion” in T2WI when ERC was preferred (*P*=0.58). By contrast, when readers did not have a general preference for one of the coil techniques, the differences in the individual scores were generally not statistically significant, either. The exceptions were better scores for “motion artifacts” (*P*<0.001) and “other artifacts” (*P*=0.03) for the FSC in T2WI and a better score for “motion artifacts” for the ERC in DWI (*P*<0.001). Supplementary Table 3 shows the pooled given mean scores for both sequences and all readers combined.

**Table 3:** Flexible surface coil and endorectal coil T2WI and DWI subjective assessment mean scores stratified by readers’ preferred coil setup in each single pair of images

	Image quality	Delineation	PZ/TZ	Distortion	Motion	Other artifacts
*FSC*						
T2WI						
FSC	1.55 ± 0.5	1.5 ± 0.6	1.49 ± 0.5	1.34 ± 0.5	1.39 ± 0.5	1.35 ± 0.5
ERC	2.45 ± 0.7	2.33 ± 0.8	2.01 ± 0.8	1.84 ± 0.9	2.09 ± 0.7	2.0 ± 1
p^a^	<0.001	<0.001	<0.001	<0.001	<0.001	<0.001
DWI						
FSC	1.85 ± 0.8	1.77 ± 0.8	1.87 ± 0.7	1.42 ± 0.7	1.40 ± 0.6	1.42 ± 0.6
ERC	2.80 ± 1.0	2.65 ± 1.1	2.65 ± 1.1	2.47 ± 1.2	1.70 ± 1	2.17 ± 1.1
p^a^	<0.001	<0.001	<0.001	<0.001	0.11	<0.001
*ERC*						
T2WI						
FSC	2.00 ± 0.7	1.80 ± 0.8	1.73 ± 0.8	1.34 ± 0.7	1.55 ± 0.8	1.44 ± 0.7
ERC	1.31 ± 0.5	1.30 ± 0.5	1.23 ± 0.4	1.27 ± 0.6	1.39 ± 0.5	1.29 ± 0.7
p^a^	<0.001	<0.001	<0.001	0.58	0.47	0.03
DWI						
FSC	2.85 ± 0.8	2.74 ± 0.9	2.82 ± 0.8	2.24 ± 1.1	1.58 ± 0.9	1.54 ± 0.8
ERC	1.63 ± 0.7	1.66 ± 0.7	1.54 ± 0.7	1.44 ± 0.6	1.16 ± 0.4	1.25 ± 0.6
p^a^	<0.001	<0.001	<0.001	<0.001	<0.001	<0.001
*No preference*						
T2WI						
FSC	1.47 ± 0.5	1.47 ± 0.6	1.43 ± 0.6	1.23 ± 0.5	1.29 ± 0.5	1.29 ± 0.5
ERC	1.54 ± 0.6	1.52 ± 0.6	1.39 ± 0.6	1.29 ± 0.6	1.5 ± 0.6	1.45 ± 0.7
p^a^	0.19	0.34	0.44	0.39	<0.001	0.03
DWI						
FSC	2.44 ± 1.1	2.35 ± 1.1	2.39 ± 1.1	2.04 ± 1.0	2.02 ± 1.1	1.95 ± 1.0
ERC	2.28 ± 1.0	2.20 ± 1.0	2.17 ± 1.0	2.06 ± 0.9	1.76 ± 0.9	2.07 ± 1.0
p^a^	0.22	0.29	0.09	0.73	<0.001	0.38

*PZ/TZ* differentiation of peripheral zone and transition zone; *T2WI* T2-weighted image; *DWI* diffusion-weighted image; *FSC* flexible surface coil; *ERC* endorectal coil; *a* Mann–Whitney *U* test; Lower score indicates better result

#### Readers consistency

The intrareader agreement, i.e. the consistency of given scores for duplicate FSC cases (Table 4), was moderate for four readers (*k:* 0.42, 0.42, 0.48, and 0.53), substantial for one reader (*k* = 0.62), and almost perfect for one reader (*k* = 0.90).

**Table 4:** Intrareader agreement: consistency of given scores for duplicate flexible surface coil examinations

Reader	*k*
R1	0.42
R2	0.90
R3	0.53
R4	0.42
R5	0.62
R6	0.48

*k* = Cohen’s kappa

### Assessment of predictors of preferred sequence

Patients’ weight was the only weak predictor of a preference for the ERC acquisition rather than for the FSC acquisition (*p* = 0.04). No associations were found between coil preference and patients’ age, PSA, prostate volume, treatment, prior experience with ERC acquisition, or years of MR imaging experience in multivariate logistic regression analysis (Table 5).

**Table 5:** Multivariate logistic regression analysis

Parameter	OR	95% CI	p
FSC cases			
Age (years)	1.15	0.82–1.62	0.31
PSA (ng/nl)	1.05	0.96–1.16	0.44
Prostate volume (cc)	1.01	0.96–1.06	0.44
Bodyweight (kg)	1.13	0.92–1.39	0.06
ERC cases			
Age (years)	0.86	0.61–1.20	0.38
PSA (ng/ml)	0.90	0.71–1.15	0.44
Prostate volume (cc)	1.04	0.89–1.14	0.23
Bodyweight (kg)	0.87	0.61–0.89	0.04
Treatment			
Active Surveillance	0.99	0.98–1.01	0.91
PPI Brachytherapy	0.94	0.20–4.38	0.87
Reader			
Prostate MRI experience (years)	0.89	0.44–1.51	0.64
Experience with ERC	0.76	0.48–1.41	0.08

Positive outcome is the option for the flexible surface coil. Parameters of each group (flexible surface coil cases and endorectal coil cases) were included independently. Results show that readers were less likely to prefer the FSC as the bodyweight of men undergoing MRI with an ERC increased. As FSC and ERC cases were matched based on bodyweight category, the average weight of the paired FSC case was also increasing

*FSC* flexible surface coil; *ERC* endorectal coil; *OR* odds ratio; *CI* confidence interval; *p* probability; *PSA* prostate-specific antigen; *PPI* permanent prostatic implant

## Discussion

The choice of the optimal receiver coil technique in mpMRI of the prostate is still a matter of debate. While many institutions prefer ERC, mainly due to the possibility to achieve a higher SNR when positioning the coil in close proximity to the gland [5], others question the benefits in image quality and diagnostic performance and emphasize disadvantages of ERC like increased costs, scanning time, patient discomfort [9, 10], and artifacts [23]. Our results suggest there is no significant difference in image quality for FSC and ERC.

Although there was a strong individual preference in the perceived overall quality for images acquired with one or the other receiver coil, the pooled estimate from all six radiologists was balanced in the overall preference for both assessed sequences combined. These findings support the thesis that personal affinity for one of the two coil setups may be subject to training or habituation, which is a major limitation of many of the published studies on this topic as readers often came from the same or closely connected institutions where usually one particular coil technique is routinely applied. This may be one of the reasons for the contradictory results in the literature [6–8, 10, 12, 15]. We included readers from six different centers in four countries so that preferences due to habituation could be reduced. However, multivariate logistic regression analysis revealed only a weak association of readers’ experience with ERC and choice of preferred image data set that was not statistically significant. This may in part be due to three readers having had experience with both coil setups.

Analysis of the individual sequences revealed a pooled preference for the FSC in T2WI and for the ERC in DWI. These results are in agreement with the studies by Barth et al. [9] and O’Donohoe et al, [13] who compared the quality of T2WI and DWI obtained with an ERC to those acquired using a pelvic phased-array coil or a wearable pelvic coil that seats nearer to the prostate, respectively. In both studies the overall quality was similar for the coils that were compared, but with a tendency for better DWI obtained using the ERC. A possible explanation for the slight preference for an ERC in DWI in contrast to T2WI might be the inherently lower SNR of DWI owing to the diffusion weighting and long echo times [24] such that this sequence benefits more from the close vicinity of an ERC to the organ. Another reason may be that ERCs displace rectal gas, which can also improve the quality of DWI.

Another very important aspect in quality assessment of MR images are artifacts that can significantly impair visibility and therefore influence the overall valuation of the images. In our evaluation readers noticed slightly fewer motion artifacts in DWI using the ERC which might have contributed to the overall affinity for this coil setup in DWI. ERC coils largely immobilize the prostate in the lower pelvis and thus artifacts from rectal peristalsis might be less frequent. However, there is emerging evidence that administration of antispasmodics can reduce the occurrence and intensity of motion artifacts in prostate MRI which might be useful for non-ERC examinations [25–27]. On the other hand, there was more distortion of the prostate, more motion artifacts, and other artifacts when the ERC was utilized. These findings may reflect the fact that ERC MR images are generally prone to signal inhomogeneity due to the non-uniform reception profile [11], susceptibility artifacts as a result of the direct interface of soft tissue and air or liquids in the ERC, and anatomical distortion [28]. These observations have been confirmed by several published studies [9, 10, 13]. Different correction algorithms have been developed to compensate signal inhomogeneity, but these may lead to noise level variation and an increase of acquisition time [11, 12, 29]. It has to be considered that rectal gas if present may lead to similar susceptibility artifacts as gas in ERC.

In contrast to the results of our study, other groups found ERC imaging not only to be qualitatively superior to non-ERC imaging but also reported better diagnostic performances with this approach [6, 7, 30]. However, it should be noted that these studies had a limited number of readers with the risk of a habituation effect and may have used scanning parameters for the two coil setups such as in-plane resolution, scan times, number of excitations, slice thickness, and b values, that favored the ERC setup. In a recent study in which scanning parameters were kept constant, pelvic phased-array coil images were perceived to have lower quality but the diagnostic performance was similar to scans obtained with an ERC [10]. Another study reported better image quality for a pelvic phased-array coil, again with similar PCA detection rates in comparison to an ERC scan [14].

Our quantitative quality analysis revealed higher SNR for the ERC compared to the FSC for all parts of the prostate, including PCA lesions, which is to be expected as a result of the proximity of the ERC to the gland, as shown in other studies [5, 13, 30]. In contrast, one study reported a higher SNR for pelvic phased-array coil DWI images and similar SNR for pelvic phased-array coil and ERC in T2WI, which is likely accounted for by a higher number of excitations for the pelvic phased-array coil images though [9].

The CNR was higher for differentiation of PZ and TZ and for differentiating PCA lesions and benign prostate tissue when the ERC was utilized, but the difference was not statistically significant for the latter. This is an important observation since tumor differentiation from benign tissue is the crucial task of mpMRI. It is noted that the SNR of PCA lesions is collectively higher than the SNR of the PZ and TZ. These tumors were probably closer to the receiver coil than the standardized ROIs that were utilized for the PZ/TZ measurements. Intrareader agreement in our study was within the range of previously published results.

In multivariate logistic regression analysis bodyweight was the only weak predictor for the choice of the preferred coil, indicating an advantage for the ERC with increasing bodyweight. This observation is not surprising as the benefit of having the ERC close to the prostate likely increase with larger patients, where there is a larger distance between the FSC and the gland.

Our study has limitations. First, we focused on the evaluation of image quality, but we did not assess diagnostic performance. However, appropriate image quality is the essential prerequisite for adequate and correct PCA diagnosis [31]. Second, blinding of the study sequences for the readout was not possible as parts of the ERC will always be visible on images. Possibly, readers could have inherent biases in favor or against one particular coil setup which may have influence on their quality assessment. We tried to mitigate this issue by defining six different objective, independent quality criteria. Third, we did not scan the same patients using FSC and ERC, but patients were matched for weight, age, prostate volume, and PSA value. Fourth, although the readers came from six different centers, all imaging was performed in one center, limiting the variation in image acquisition. Fifth, rectal loading was not accounted for.

In conclusion, although readers have strong individual preferences, comparable subjective image quality can be obtained for prostate MRI at 3 T with an ERC and the novel FSC, that can be placed in close proximity to the prostate. ERC imaging might be particularly valuable for sequences with inherently lower SNR such as DWI and larger patients whereas the FSC is generally preferred in T2WI where readers appreciated less image distortion, less motion, and other artifacts. FSC imaging generates a lower SNR than with an ERC.

## Electronic supplementary material

Below is the link to the electronic supplementary material.

Supplementary material 1 (DOCX 21 kb)
