# Supplementary material for: Quality Comparison of 3 Tesla multiparametric MRI of the prostate using a flexible surface receiver coil versus conventional surface coil plus endorectal coil setup
Source: Abdom Radiol (NY). 2020 Jul 21;45(12):4260–70. doi: 10.1007/s00261-020-02641-0 (PMC7716937; doi:10.1007/s00261-020-02641-0)
Supplement: Supplementary file 1 — Supplementary material 1 (DOCX 21 kb) [file 261_2020_2641_MOESM1_ESM.docx]

Supplementary Table 1: Baseline characteristics of patient sample.

| Patient Characteristics | | FSC | ERC | p-value^a^ |
| --- | --- | --- | --- | --- |
| Bodyweight (kg) | Median (range) | 84.9 (75.2–95.6) | 83.6 (74.5–97.1) | 0.69 |
| Age (years) | Median (range) | 66 (63–70) | 67 (63–72) | 0.70 |
| Prostate Volume (ml) | Median (range) | 48.1 (30.8–79.1) | 45.9 (29.8–78) | 0.71 |
| PSA (ng/ml) | Median (range) | 6.4 (4.4–8.4) | 5.9 (4.5–8.48) | 0.61 |
| FSC = flexible surface coil; ERC = endorectal coil; PSA = prostate specific antigen; a = Mann-Whitney U test | | | | |

Supplementary Table 2: Count (percentage) of the choice for the generally preferred MRI examination and sequences for each reader.

|  | | Technique | | |
| --- | --- | --- | --- | --- |
| Reader | | FSC | Either | ERC |
| 1 | Entire study | 73 (36.5) | 100 (50) | 27 (13.5) |
|  | T2WI | 52 (52) | 38 (38) | 10 (10) |
|  | DWI | 21 (21) | 62 (62) | 17 (17) |
| 2 | Entire study | 50 (25) | 59 (29.5) | 91 (45.5) |
|  | T2WI | 34 (34) | 49 (49) | 17 (17) |
|  | DWI | 16 (16) | 10 (10) | 74 (74) |
| 3 | Entire study | 40 (20) | 68 (34) | 92 (46) |
|  | T2WI | 26 (26) | 47 (47) | 27 (27) |
|  | DWI | 14 (14) | 21 (21) | 65 (65) |
| 4 | Entire study | 30 (30) | 27 (27) | 43 (43) |
|  | T2WI | 20 (40) | 18 (36) | 12 (24) |
|  | DWI | 10 (20) | 9 (18) | 31 (62) |
| 5 | Entire study | 24 (24) | 35 (35) | 41 (41) |
|  | T2WI | 15 (30) | 22 (44) | 13 (26) |
|  | DWI | 9 (18) | 13 (26) | 28 (56) |
| 6 | Entire study | 45 (45) | 40 (40) | 15 (15) |
|  | T2WI | 27 (54) | 22 (44) | 1 (2) |
|  | DWI | 18 (36) | 18 (36) | 14 (28) |
| All | Entire study | 262 (29.1) | 329 (36.6) | 309 (34.3) |
|  | T2WI | 174 (38.7) | 196 (43.6) | 80 (17.8) |
|  | DWI | 88 (19.6) | 133 (29.6) | 229 (50.9) |
| FSC = flexible surface coil ; ERC = endorectal coil; T2WI = T2-weighted image; DWI = diffusion-weighted image | | | | |

Supplementary Table 3: Pooled Flexible surface coil and endorectal coil quality assessment mean scores.

|  | FSC | ERC | p-value^a^ |
| --- | --- | --- | --- |
| Image Quality | 2,07 ± 0,92 | 1,95 ± 0,89 | 0.23 |
| Deleneation | 1,99 ± 0,95 | 1,90 ± 0,89 | 0.11 |
| PZ/TZ | 2,01 ± 0,95 | 1,77 ± 0,88 | < 0.001 |
| Distortion | 1,66 ± 0,91 | 1,66 ± 0,88 | 0.59 |
| Motion | 1,53 ± 0,78 | 1,59 ± 0,74 | < 0.001 |
| Other Artifacts | 1,49 ± 0,75 | 1,65 ± 0,90 | < 0.001 |

PZ/TZ = differentiation of peripheral zone and transition zone; FSC = flexible surface coil; ERC = endorectal coil; a = Mann-Whitney U test; Lower score indicates better result
